# Frantz Tumor (Solid Pseudopapillary Neoplasm) Mimicking an Infected Pancreatic Pseudocyst: A Case Report

**DOI:** 10.7759/cureus.103308

**Published:** 2026-02-09

**Authors:** Flaviano Junqueira, Murilo Henrique Pedrão Ferreira, Leticia Mancilla Lourenço, Everson Artifon

**Affiliations:** 1 Medicine, University of São Paulo, São Paulo, BRA; 2 Surgery, University of São Paulo, São Paulo, BRA

**Keywords:** case report, frantz tumor, pancreas, pancreatic pseudocyst, solid pseudopapillary neoplasm

## Abstract

Frantz tumor, or solid pseudopapillary neoplasm (SPN), is a rare pancreatic tumor of low malignant potential that predominantly affects young women. Its clinical presentation is often nonspecific, and the differential diagnosis from other cystic pancreatic lesions, such as pseudocysts, represents a significant challenge. We report the case of a 31-year-old previously healthy woman who presented with abdominal pain, initially diagnosed and treated as an infected pancreatic pseudocyst. Following the initial surgical intervention and antibiotic therapy, the persistence of imaging findings and subsequent histopathological results led to the definitive diagnosis of a Frantz tumor. The patient subsequently underwent a curative-intent pancreatoduodenectomy. This case highlights the difficulty in distinguishing cystic pancreatic lesions. The patient’s initial presentation, with a large fluid collection, gas foci, and elevated inflammatory markers, strongly suggested an infectious process, such as a complicated pseudocyst. Only histopathological examination of the surgical specimen enabled the correct diagnosis of the underlying neoplasm. SPN may show cystic and hemorrhagic degeneration, mimicking other lesions. Frantz tumor should be considered in the differential diagnosis of cystic pancreatic lesions in young women, even when the initial presentation suggests an inflammatory or infectious process. Histopathological confirmation is essential for proper management, which consists of complete surgical resection and is associated with an excellent prognosis.

## Introduction

Solid pseudopapillary neoplasm (SPN) of the pancreas, also known as Frantz tumor, is a rare clinicopathological entity, accounting for only 1-2% of all exocrine pancreatic tumors [1,2]. First described by Virginia Frantz in 1959, this neoplasm characteristically affects young women, with an average age at diagnosis between 25 and 35 years, and is extremely rare in men [3].

Although classified by the World Health Organization (WHO) as a neoplasm with low malignant potential, SPN has an excellent prognosis after complete surgical resection, with five-year survival rates exceeding 95% [4]. The clinical presentation is usually insidious, with nonspecific symptoms such as abdominal pain or discomfort, or the palpation of an abdominal mass. In many cases, the diagnosis is incidental during imaging studies performed for unrelated reasons [5].

Radiologically, Frantz tumors typically appear as a well-defined, encapsulated mass with variable proportions of solid and cystic components, which may exhibit areas of hemorrhage or necrosis [1]. This heterogeneity can make the differential diagnosis challenging, including other cystic pancreatic lesions such as serous and mucinous cystadenomas, cystic neuroendocrine tumors, and, notably, pancreatic pseudocysts [6]. Confusion with pseudocysts is particularly relevant, as they are the most common cystic lesions of the pancreas and are generally associated with a history of pancreatitis.

This report describes a challenging case of a 31-year-old female patient whose initial diagnosis was that of an infected pancreatic pseudocyst, treated with drainage and antibiotic therapy, but whose subsequent investigation revealed a Frantz tumor. The purpose of this work is to emphasize the importance of including SPN in the differential diagnosis of complex cystic pancreatic lesions in young women, even in the absence of a typical clinical or radiological presentation.

## Case presentation

A 31-year-old previously healthy female patient sought medical care on the day of presentation with complaints of abdominal pain. Initial investigation with endoscopic ultrasound revealed a solid-cystic lesion in the pancreas. Fine-needle aspiration biopsy was negative for malignancy, although with scarce material. The clinical picture evolved with worsening pain, nausea, and vomiting, prompting hospitalization on day 3. Imaging studies (CT and MRI) demonstrated a large pancreatic collection with an air-fluid level and gaseous foci, suggestive of an infected pseudocyst. The patient was treated with broad-spectrum antibiotic therapy (ceftriaxone and metronidazole) and subsequently underwent percutaneous drainage on day 10, with culture of the material revealing *Streptococcus anginosus*. The antimicrobial regimen was adjusted to teicoplanin and later to ciprofloxacin. Despite initial clinical improvement and reduction in collection volume on follow-up imaging, the patient underwent gastric derivation of the pseudocyst with cholecystectomy on day 30, due to the complexity of the case. Culture of the surgical material identified *Staphylococcus epidermidis,* and histopathological analysis of the cyst wall and adjacent pancreas showed fibrosis and acute-on-chronic inflammation (acute cholecystitis in the gallbladder). Surprisingly, the definitive histopathological analysis of the surgical specimen, whose result was released on day 60, diagnosed SPN (Frantz tumor).

**Figure 1:**
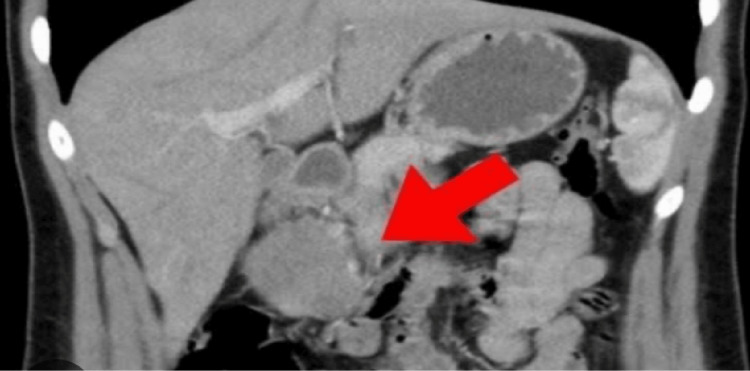
Solid pseudopapillary neoplasm of the pancreatic head on the coronal CT scan

This coronal CT image (Figure 1) highlights an SPN of the pancreas, indicated by the arrow, located in the pancreatic head. The radiological appearance demonstrates a well-defined mass with mixed solid-cystic components, a pattern frequently described in this low-grade malignant tumor that predominantly affects young women. This figure serves as a visual reference for the lesion’s typical anatomical position and structural characteristics within the pancreatic region.

With the oncological diagnosis established, the patient was staged. Tumor markers (CA 19-9, CEA) were normal. After surgical risk assessment, she underwent gastroduodenopancreatectomy with retroperitoneal lymphadenectomy and Roux-en-Y reconstruction on Day 110, a procedure that occurred without complications.

Postoperative histopathological and immunohistochemical findings

Histopathological examination of the surgical specimen revealed an SPN characterized by pseudopapillary architecture, uniform neoplastic cells with eosinophilic cytoplasm, oval nuclei with nuclear grooves, and areas of hemorrhage and cystic degeneration. Surgical margins were free of tumor, and no lymph node metastasis was identified.

Immunohistochemical analysis demonstrated nuclear positivity for β-catenin and cyclin D1, with positivity for progesterone receptor and cytokeratin AE1/AE3. The Ki-67 proliferation index was low (<1%). Neuroendocrine markers, including chromogranin A and synaptophysin, were negative. This immunophenotypic profile is consistent with an SPN of the pancreas.

**Figure 2:**
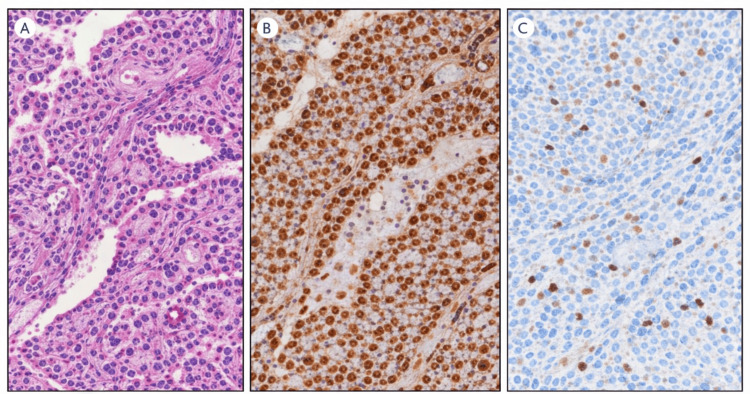
Histopathological and immunohistochemical features of solid pseudopapillary neoplasm of the pancreas. (A) Hematoxylin and eosin staining showing pseudopapillary architecture with uniform tumor cells. (B) Nuclear accumulation of β-catenin on immunohistochemistry. (C) Low proliferative activity demonstrated by the Ki-67 labeling index.

As shown in Figure 2, we observe the complete anatomical specimen obtained during the gastroduodenopancreatectomy procedure, which demonstrates the characteristic gross pathological features of an SPN. The resected tissue displays the typical heterogeneous appearance with both solid and cystic components, along with areas of hemorrhage and necrosis that are commonly observed in this type of pancreatic tumor.

**Figure 3:**
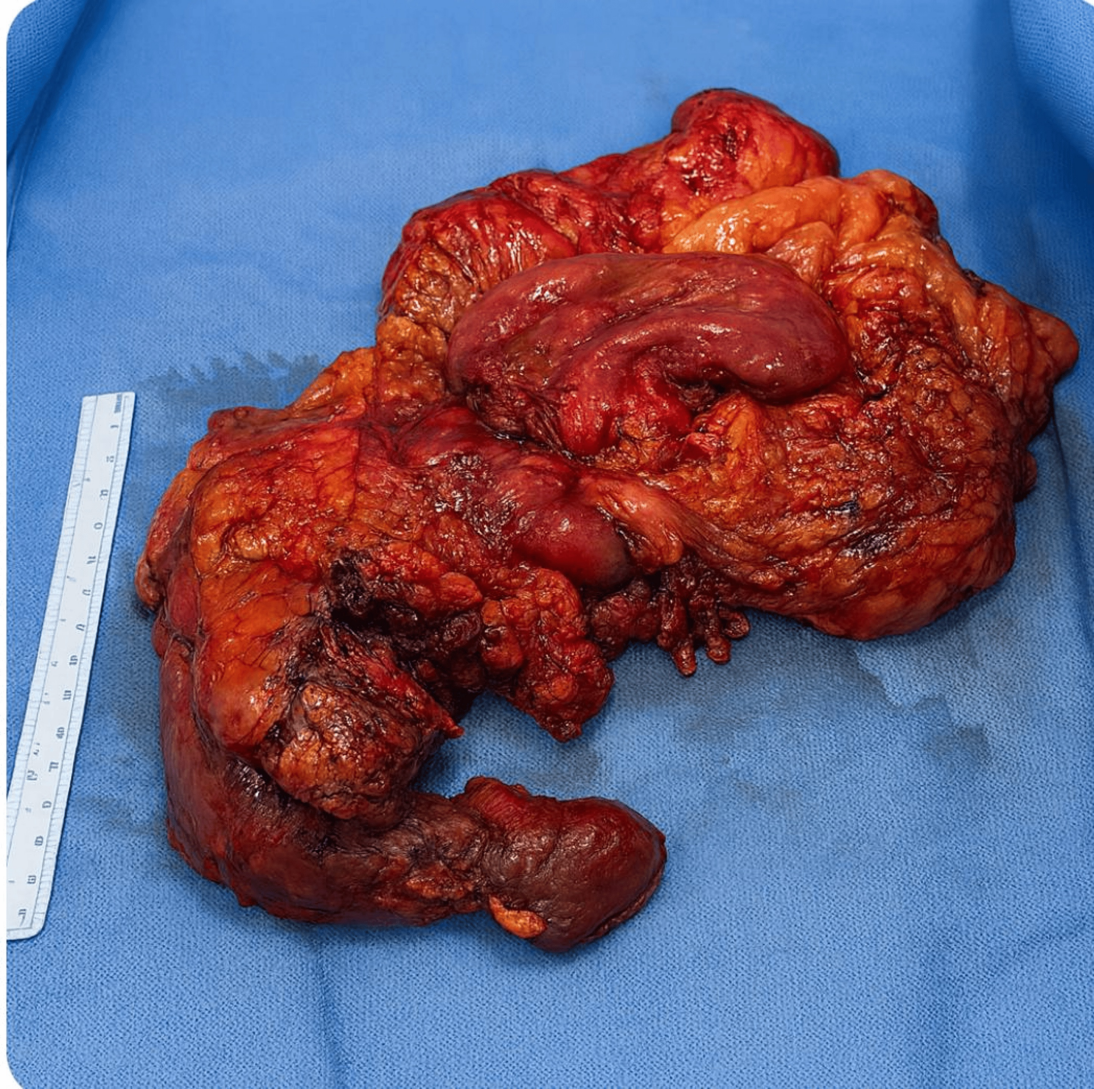
Anatomical specimen removed during gastroduodenopancreatectomy Source: Authors' own elaboration

The anatomical specimen was removed during gastroduodenopancreatectomy on day 110 (Figure 3).

**Figure 4:**
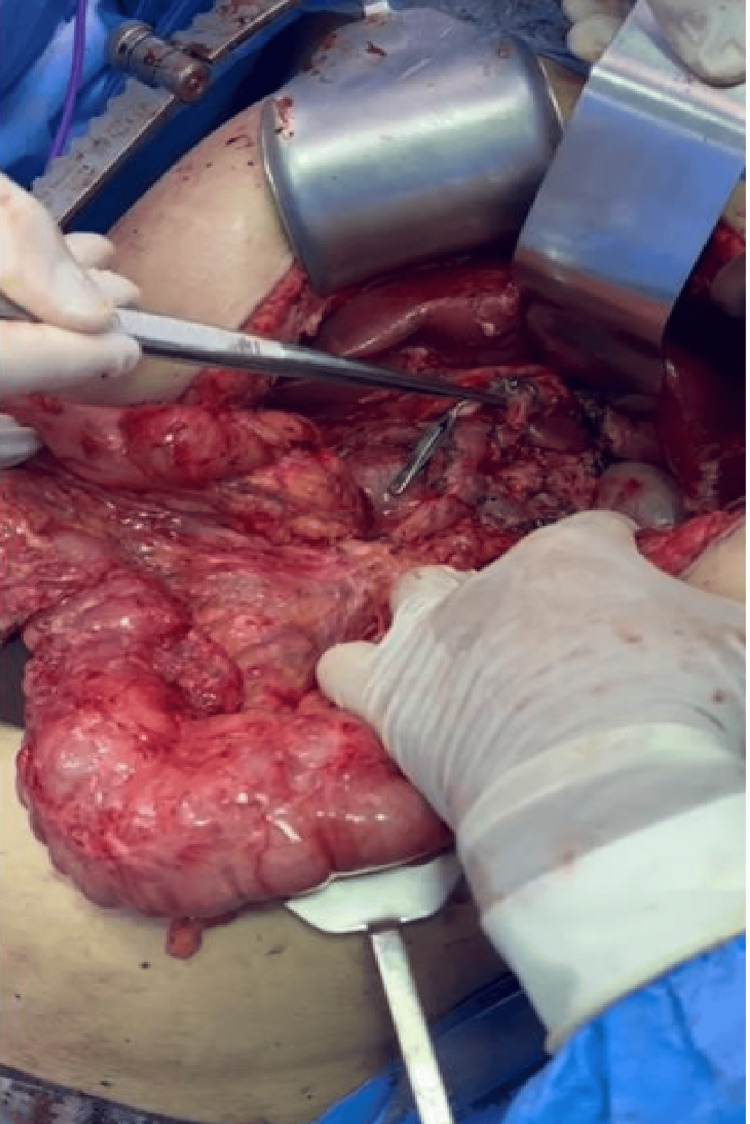
Intraoperative view showing pancreatic head of approximately 4 cm and clipped gastroduodenal artery and hepatic artery Source: Authors' own elaboration

As depicted in Figure 4, the intraoperative view during the pancreaticoduodenectomy reveals the complex anatomical relationships and the surgical technique employed for tumor resection. The procedure involved careful dissection of the pancreatic head, demonstrating the proximity of the lesion to vital structures, including the gastroduodenal artery and hepatic vessels. This intraoperative documentation illustrates the surgical approach and the technical considerations necessary for complete tumor removal.

The diagnostic course, summarized in Tables 1-2, was prolonged and marked by an initial misinterpretation of the pancreatic lesion as an infected pseudocyst. Despite early imaging and endoscopic evaluation, the overlap between inflammatory findings and cystic degeneration delayed the recognition of an underlying neoplasm. Definitive diagnosis was achieved only after surgical intervention, underscoring the limitations of minimally invasive sampling in complex cystic pancreatic lesions.

**Table 1:** Laboratory test timeline of the Frantz tumor case

Test	Date 1	Value 1	Date 2	Value 2	Normal reference values
CEA	Day 64	<0.5	Day 126	<0.5	<3.0 ng/mL
CA 19-9	Day 64	22.06	Day 126	23.19	<37.0 U/mL
CA 125	Day 64	7.2	Day 126	4.4	<35.0 U/mL
Lipase	Day 75	19	Day 158	8	10-140 U/L
Amylase	Day 75	18	Day 159	28	25-125 U/L
CRP	Day 75	235.8	Day 159	6.5	<3.0 mg/L
Leukocytes	Day 75	22,44	Day 159	8,88	4,000-11,000 cells/μL

**Table 2:** Chronological timeline of the diagnostic procedures and therapeutic interventions Source: Authors' own elaboration

Exam	Date	Relevant result
Endoscopic ultrasound	Day of presentation (Day 0)	Solid-cystic lesion in the pancreas.
Pathological anatomy (puncture)	Day 1	Negative for malignancy (scarce material)
Abdominal MRI	Day 3	Cystic formation with air-fluid level and gas (suspected infected pseudocyst)
Percutaneous drainage	Day 10	Culture: *Streptococcus anginosus*
Surgery 1 (derivation)	Day 30	Culture: *Staphylococcus epidermidis*. PA: chronic inflammation
Pathological Anatomy (Final)	Day 60	Solid pseudopapillary neoplasm (Frantz tumor)
Surgery 2 (Pancreatectomy)	Day 110	Gastroduodenopancreatectomy

Laboratory findings supported this diagnostic challenge. As shown in Table 1, conventional tumor markers remained within normal limits throughout the disease course, while inflammatory markers were markedly elevated during the infectious phase. This dissociation between inflammatory activity and stable pancreatic enzymes reinforced the atypical presentation of an SPN in this patient.

The chronological sequence of diagnostic procedures and therapeutic interventions is summarized in Table 2, which illustrates the evolution from initial presentation to definitive diagnosis and treatment.

## Discussion

A Frantz tumor is a rare neoplasm that presents a diagnostic challenge due to its variable presentation. In the reported case, the initial manifestation as a complex cystic collection, with clinical and radiological signs of infection, led to a presumptive diagnosis of infected pancreatic pseudocyst. This atypical presentation is one of the diagnostic pitfalls described in the literature. What differentiates the present case is the presence of a clinically and radiologically confirmed infected pancreatic collection, with intralesional gas and positive microbiological cultures, mimicking an infected pseudocyst. The documented superimposed infection, associated with a negative initial endoscopic biopsy and the need for multiple therapeutic interventions, masked the underlying neoplasm and delayed the definitive diagnosis until postoperative histopathological examination.

Although most SPNs present as well-defined masses with solid and cystic components, extensive cystic degeneration, hemorrhage, and necrosis may lead to a purely cystic appearance, mimicking pseudocysts or cystadenomas [7,8]. The superimposition of an infection, as evidenced by the growth of Streptococcus anginosus and Staphylococcus epidermidis and by imaging findings, further complicated the differential diagnosis. The presence of gas within a pancreatic lesion is a strong indicator of infection, usually associated with infected pseudocysts or abscesses, but rarely described in uncomplicated SPNs. It is believed that the rapid growth rate of the tumor may exceed its blood supply, leading to central necrosis and, occasionally, communication with the pancreatic duct or gastrointestinal tract, which could facilitate secondary infection [9]. The gold standard treatment for Frantz tumor, regardless of size or presence of metastases, is complete surgical resection with negative margins [4]. The type of resection depends on the location and extent of the tumor, ranging from enucleation for small lesions to more radical procedures such as distal pancreatectomy or gastroduodenopancreatectomy, as performed in our patient. The prognosis after resection is excellent, even in cases with local invasion or metastases, which are rare at the time of diagnosis (approximately 10-15%) [3,10]. This case highlights the importance of histopathological confirmation for the definitive diagnosis of pancreatic cystic lesions. The initial fine needle aspiration was inconclusive, a common problem due to the heterogeneous nature of the tumor and the possibility of sampling necrotic or cystic areas. The final diagnosis was only possible after analysis of the surgical specimen, reinforcing the role of surgery not only as a therapeutic, but also as a definitive diagnostic tool in ambiguous cases.

## Conclusions

This case report of a Frantz tumor mimicking an infected pancreatic pseudocyst highlights a significant diagnostic challenge. SPN should be maintained in the differential diagnosis of young women presenting with complex cystic lesions in the pancreas, even when signs and symptoms strongly suggest an infectious process. Complete surgical resection is the treatment of choice and offers a favorable prognosis, with histopathological confirmation being essential for adequate therapeutic planning.
